# Histopathological Images Analysis and Predictive Modeling Implemented in Digital Pathology—Current Affairs and Perspectives

**DOI:** 10.3390/diagnostics13142379

**Published:** 2023-07-14

**Authors:** Mihaela Moscalu, Roxana Moscalu, Cristina Gena Dascălu, Viorel Țarcă, Elena Cojocaru, Ioana Mădălina Costin, Elena Țarcă, Ionela Lăcrămioara Șerban

**Affiliations:** 1 Department of Preventive Medicine and Interdisciplinarity, “Grigore T. Popa” University of Medicine and Pharmacy, 700115 Iassy, Romania; moscalu.mihaela@gmail.com; 2 Wythenshawe Hospital, Manchester University NHS Foundation Trust, Manchester Academic Health Science Centre, Manchester M139PT, UK; roxana.moscalu@mft.nhs.uk; 3 Department of Morphofunctional Sciences I, “Grigore T. Popa” University of Medicine and Pharmacy, 700115 Iassy, Romania; elena2.cojocaru@umfiasi.ro; 4 Faculty of Medicine, “Grigore T. Popa” University of Medicine and Pharmacy, 700115 Iassy, Romania; mg-fr-31417@students.umfiasi.ro; 5 Department of Surgery II—Pediatric Surgery, “Grigore T. Popa” University of Medicine and Pharmacy, 700115 Iassy, Romania; 6 Department of Morpho-Functional Sciences II, Faculty of Medicine, “Grigore T. Popa” University of Medicine and Pharmacy, 700115 Iassy, Romania; ionela.serban@umfiasi.ro

**Keywords:** artificial intelligence, digital pathology, predictive modeling

## Abstract

In modern clinical practice, digital pathology has an essential role, being a technological necessity for the activity in the pathological anatomy laboratories. The development of information technology has majorly facilitated the management of digital images and their sharing for clinical use; the methods to analyze digital histopathological images, based on artificial intelligence techniques and specific models, quantify the required information with significantly higher consistency and precision compared to that provided by optical microscopy. In parallel, the unprecedented advances in machine learning facilitate, through the synergy of artificial intelligence and digital pathology, the possibility of diagnosis based on image analysis, previously limited only to certain specialties. Therefore, the integration of digital images into the study of pathology, combined with advanced algorithms and computer-assisted diagnostic techniques, extends the boundaries of the pathologist’s vision beyond the microscopic image and allows the specialist to use and integrate his knowledge and experience adequately. We conducted a search in PubMed on the topic of digital pathology and its applications, to quantify the current state of knowledge. We found that computer-aided image analysis has a superior potential to identify, extract and quantify features in more detail compared to the human pathologist’s evaluating possibilities; it performs tasks that exceed its manual capacity, and can produce new diagnostic algorithms and prediction models applicable in translational research that are able to identify new characteristics of diseases based on changes at the cellular and molecular level.

## 1. Introduction

In modern clinical practice, digital pathology has an essential role, being a technological necessity for the activity in the pathological anatomy laboratories [1]; it allows us to improve diagnostic accuracy, to reduce evaluation time and to create more efficient workflows, given that from year to year these services are facing an increasing workload in terms of the demand for analysis, the complexity of the investigations requested and response times being more and more reduced. In the UK, the demand for anatomopathological analysis grows yearly by 4.5%, and in 2020 the average response time varied between 2 weeks (50% of patients) and 4 weeks (95% of patients) [2]. Digital applications can change diagnosis methods, bringing benefits through the integrated diagnostic approach, with direct effects of improving patient care and safety and indirect effects of optimizing staff performance and reducing costs [3].

The development of information technology (IT) has majorly facilitated the management of digital images and their sharing for clinical use [4,5], and the methods to analyze digital histopathological images, based on artificial intelligence techniques and specific models, quantify the required information with significantly higher consistency and precision compared to that provided by optical microscopy [6].

In parallel, the unprecedented advances in machine learning facilitate, through the synergy of artificial intelligence and digital pathology, the possibility of diagnosis based on image analysis, previously limited only to certain specialties—radiology, cardiology, etc. [1,7]—and provide tools to identify tumors and to evaluate biomarkers with high efficiency, integrated into extensible and flexible software platforms [8,9,10].

Therefore, the integration of digital images into the study of pathology, combined with advanced algorithms and computer-assisted diagnostic techniques, extends the boundaries of the pathologist’s vision beyond the microscopic image and allows the specialist to use and integrate his knowledge and experience adequately [1,11].

Digital pathology is defined as utilizing a computer to visualize histopathological images in Whole-Slide Imaging (WSI) format, obtained by the high-resolution scanning of tissue samples, as well as to store and to analyze the images and their associated meta-data for diagnostic purposes through general techniques of “pattern recognition”, particularly through “deep learning” techniques. A related concept, more and more popular, is that of “computational pathology”, which regards the computerized analysis of large imagistic data volumes (“big-data”) and meta-data, acquired through multiple sources, in order to extract relevant biological and clinical features and to identify patterns, thus allowing us to establish diagnosis and provide accurate forecasts [12].

## 2. Data Sources and Literature Search Strategy

We conducted a search in PubMed on the topic of digital pathology and its applications to quantify the current state of knowledge. In the first stage, our goal was to identify all the articles published in the field; therefore, we used in the search the keywords “digital pathology”, “computational pathology” and “digital microscopy”, in the article’s title or summary; the search provided 2475 results, published between 1984 and 2022. Of these, before 2000, 37 articles were published, i.e., 1.5% of the total, with the rest of the papers being published after 2000 (respectively, 2438—98.5% of the total). During the last 5 years (2017–2022), 1678 articles have been published, which represents almost two-thirds of the total published papers (67.8%)—clearly, at present, this research direction has an accelerated dynamic, and many researcher teams around the world report significant results. Among the published papers, 281 articles were reviews, systematic reviews or meta-analyses and were not further included. A synthesis of the search results by different keywords located only in the title or summary (to obtain the most relevant results) is presented below (Table 1).

We found that the number of articles discussing applications integrated in information systems and dedicated to image analysis is quite low (28—1.7%). This was also true for articles discussing image analysis software, without the specific functions of information systems (99—5.9%)—such applications are less complex than the information systems and are therefore easier to develop, which explains the more frequent concerns about them. The using of modern WSI technology in histopathology is very popular in the scientific environment—a quarter of the articles published in the last 5 years (429—25.6%) are dedicated to this topic, with its applications and possible improvements. Among the applications of digital pathology in clinical diagnosis, the most frequent research is directed towards segmentation techniques (181 articles—10.8%) and, respectively, biomarker evaluation (115 articles—6.9%). There are also numerous articles discussing the applications of digital pathology in education and training (358—21.3%), with those dealing with quality control being less common (27—1.6%). Regarding the informatics methods used in digital pathology, a great share is represented by machine learning techniques (1063 articles—63.3%) and, within it, by deep learning (407 articles—24.3%); statistical approaches, which also represent a viable solution, are less popular (92 articles—5.5%); they are usually only involved in assessing the performance degree of the processing algorithms, calculated through similarity measures (62 articles—3.7%).

## 3. Whole-Slide Imaging

The WSI technique provides benefits through the increased amount of visual information made available for the anatomo-pathologist, which is otherwise impossible to extract and interpret. Through this technique, firstly described by Wetzel and Gilbertson in 1999, the entire tissue specimen is converted into digital format through scanning. The procedure begins with a global scan of the entire specimen at low resolution, after which the obtained image is divided into small sections (called “patches”) that are scanned at maximal resolution. For each patch, several scans are performed, progressively increasing the resolution and the magnitude, and the resulting images are stored in a pyramidal structure—the low-resolution ones being placed at the top, and the high-resolution ones being placed towards the base and possibly archived [13]. The global image also contains identifiers: patient’s name, barcodes, preparation details, etc.

The technique therefore provides a package of high-resolution images (at least 100 k × 100 k), in color, without anatomical orientation, which can be visualized at different magnitude scales (×4, ×10, ×20) and from different angles (because each section has a well-defined thickness and, depending on the focus plane, will generate different images)—in a way somehow similar to the navigation in Google Maps [4]. Modern scanners are additionally equipped with optical systems for autofocus and focal plane selection, allowing for the accurate recording of 3D tissular morphology, similar to digital images. Such systems use several focus points located in different focal planes, adapted to the sections’ thickness, allowing the tissues’ clear representation—this is equally a difficulty, since some areas of the analyzed sample may remain out of focus. The solution is to automatically identify such regions and to add additional focus points by hand [14,15].

This technique has to deal with a significant difficulty: when slicing the tissue sample and extracting images, the spatial relationship between the serial sections, in thickness (the third dimension), is destroyed; this relation is necessary for the in-depth investigation of specific tissue areas, identified as regions of interest (ROI). It is therefore necessary to re-align the images to be investigated on the third axis of coordinates, by translation and rotation, in order to reconstruct in depth the 2D target region of interest into the shape of a 3D volume of interest—this process is called “image registration”.

Another difficulty is that the available software applications do not provide specific visualization and computational tools for WSI histopathological images and very large 2D imagery data [10].

The next step is the 3D WSI imagery [16], which allows scanners to cross objects, surfaces or even areas of the human body, obtaining in-depth images sequences (z-stack) to be converted into a 3D digital representation of the object, which is much more relevant for tissues visualization and clinical diagnosis. Nowadays, 3D reconstructions are performed using serial sections in the tissue sample, made with robotic microtomes at thicknesses between 4 and 6 μm; each section is prepared on glass slides in the classical manner and is colored with routine procedures as consistently and coherently as possible, after which the images are scanned, recorded, segmented, interpolated, and rendered in volume. This workflow puts pressure on researchers to develop more effective intelligent techniques for detecting, segmenting, analyzing, and searching 3D packages of WSI images. The computational complexity issue will exacerbate when the multi-spectral imaging techniques become widely available; it will be possible to investigate multiple tissue biomarkers because each tissue specimen will be represented on several wavelengths, in order to more accurately characterize the chromatic properties of the colors and markers documented by hundreds of images.

Current studies show that the quality of the images obtained through WSI scanning significantly influences the physician’s performance and the diagnosis accuracy [14,15,17].

### 3.1. Standard Steps in the Automatic Analysis of Histopathological Images [7]

#### 3.1.1. Pre-Processing

The source images are digital images acquired through the WSI technique, with a high resolution and large size (100,000 × 100,000 pixels); they are sampled in order to extract small-size patches (between 256 × 256 and 960 × 960 pixels) that are processed individually for features extraction and classification, in order to detect the regions of interest. A major problem at this stage is the correct identification of regions of interest, since the patches extracted from the source image are individually analyzed and classified, thus facilitating the appearance of false-positive cases. One possible solution is to make the decision based on the local average, so that a region on the specimen is classified as a region of interest only if multiple other regions of interest were identified already in its neighborhood; the disadvantage of this approach, however, is that it facilitates the occurrence of false-negative cases, since the small regions of interest tend to be omitted (e.g., isolated tumor cells).

#### 3.1.2. Processing through Machine Learning Algorithms 

(Supervised, unsupervised, semi-supervised or multiple instance learning) to interpret the extracted features. Supervised learning algorithms aim to calculate a function that assigns new input images to the correct category (e.g., “cancer”) using old, annotated images on which the function was trained; examples of working methods are support vector machines, random forest, or convolutional neural networks. Unsupervised learning algorithms aim to calculate a function that describes the internal structures and regions of similarities in new input images, using techniques such as dimensions reduction through principal components analysis, clustering (k-means), anomaly detection, etc., without using reference images for this purpose.

The other two categories of algorithms are derived from the former and combine their methods.

### 3.2. The Deep-Learning Algorithms

A particular category of algorithms with very good results in image analysis is deep-learning algorithms, which are trained using multi-layered neural networks and very large data volumes. The data are entered into an input layer of the network and are sequentially and hierarchically processed in an increasingly complex manner on each layer of the network, then optimized and organized in the way the human brain works. The deep multi-layered neural networks designed specifically for image analysis are convolutional (ConvNet/CNN—Convolutional Neural Networks); they contain a kernel/filter to convolve an image and to extract useful features necessary to differentiate it [12].

The systems that perform such operations (PRIA—Pattern Recognition Image Analysis) generally use a common workflow to identify and quantify the regions of interest on digital histopathological images; the users must provide first representative images for each tissue category, to be taken as references; during the training stage, the application identifies the unique spatial and spectral features that characterize the pixels in each reference image, and in the working stage new images are analyzed according to these landmarks in order to identify, through segmentation, the relevant pixels. The main manufacturers of such applications are Aperio Technologies—GENie, Cambridge Research and Instrumentation, Definiens and Visiopharm.

The performances of such algorithms depend on the quality, and especially on the size, of the imagistic database used for training; at present, however, such databases are not very detailed, since the annotation of anatomopathological images with the features relevant for diagnosis is carried out only by the specialist physician and almost always globally at the clinical case level— instead, the machine learning algorithms need images annotated at the patch and even at the pixel level, with all the possible features to follow, which represents a huge human workload. There are several public databases with high-resolution, annotated WSI images that can be used for training (The Cancer Genome Atlas—TCGA, which contains over 10,000 WSI images of different types of cancer, Genotype-Tissue Expression—GTEx, which contains over 20,000 WSI images with different types of tissue, the Medical Image Computing and Computer-Assisted Intervention Society—MICCAI, specialized in brain tumors), but resources of this type are still insufficient. An interesting practice in medical imaging is to organize open competitions for pathologists and automatic analysis tools, on public databases, for testing the level of competence and efficacy; the list of all such competitions is available on the Grand Challenge website (https://grand-challenge.org/challenges/ accessed on 25 May 2023).

## 4. Current Applications of Digital Pathology

### 4.1. Clinical Diagnosis

Digital histopathological images are useful to establish diagnosis by tissue examination after performant scanning with special methods like Whole-Slide Imaging (WSI) [18,19,20]. Current research has identified a percentage of concordance of between 89 and 99% between this diagnostic method and the traditional methods, which use slides and optical or electronic microscopy [21,22], so that nowadays there are pathological anatomy laboratories that have given up completely (e.g., General Pathology Laboratory—Kalmar County Hospital, Sweden) or partially (e.g., Ohio State University) at using classical investigation on slides [23,24].

#### Automatic Image Analysis Includes

CAD (Computer-Assisted Diagnosis) systems are more accurate and consistent than classical visual interpretation, already being successfully used to identify the regions of interest on the investigated specimen, as well as other details: specific staining (estrogen or progesterone receptors, HER2/neu evaluation in breast cancer, Ki67 evaluation in carcinoid tumors), tumor staging, mitosis presence, vascular invasion presence and quantification, etc.

The success of these methods depends on the quality of image standardization, carried out during their acquisition, although other problems can arise and influence the decision process [19,25]. The WSI technique facilitated the development of these methods—otherwise, the pathologist could not process the entire image due to the very large amount of visual information; instead, he would select one or more regions of interest and establish the diagnosis by analyzing only these regions. When the entire image is available through WSI, the regions of interest can be automatically selected through image processing methods.

CBIR (Content-Based Image Retrieval) systems aim to identify images that are similar to a target image, or that correspond to certain selection criteria. They are especially useful to diagnose rare cases since they provide relevant histopathological images, which can be used as a reference. The search can be conducted both by supervised and unsupervised methods—the supervised methods are more accurate, because they require specifying some features possessed by the searched image and selecting the results by calculating a similarity measure. The working speed of such systems, which use very large imagery databases, is optimized through the techniques features reduction (PCA), quick search (kd-tree, hashing) or deep-learning (e.g., SMILY—Similar Medical Image Like Yours) [26].

Automatic Image Analysis Is Successfully Used in Solving a Wide Range of Problems Faced by the Anatomist-Pathologist, among Which We Can Mention

Image standardization by eliminating the color variations. Immunohistochemical staining is a common procedure necessary for detecting the biological features of different types of tumors, to make prognoses of evolution and to select the appropriate therapy in oncologic patients [27,28,29].

Color variations occur frequently in digital histopathological images due to objective reasons: the use of coloring agents from different batches or manufacturers, variations in the thickness of tissue sections, different technical peculiarities of scanning devices, or different protocols for histological specimens staining [7,12,30]. Their existence raises major difficulties in the correct interpretation of images, both for the anatomo-pathologist as well as for the computerized systems for decision assistance, so it is necessary to reduce or eliminate such variations using color normalization techniques. The range of available techniques is wide, and many studies have been conducted to compare their quality performance or to propose new methods [31,32,33,34,35,36,37,38]. A general classification of color normalization techniques groups them into three major categories [19]: global normalization, normalization after color separation through supervised learning, and normalization after color separation through unsupervised learning.

Global normalization is based on image histogram equalization and contrast modification, which, however, can produce artifacts on the resulting image because the contrast is artificially altered; a better method is to modify the color intensity in the source image by fitting it into an average variation range set as benchmark, while the overall image contrast and the variations in intensity are preserved [39]. The color separation through supervised learning is achieved through deconvolution methods, and the color separation through unsupervised learning is achieved through statistical or mathematical methods (independent component analysis (ICA), non-negative matrix factorization (NMF), etc.).

Nuclear segmentation is used for the morphometric identification of nuclei in various anatomical regions and the segmentation of other histological structures [40]. It is one of the most-studied problems of artificial intelligence [41]; it aims to obtain the final nuclear shapes and to separate the nuclei in touch, in order to achieve the individual segmentation of the nuclei in the image. It is the basis of a new histological specialization, namely quantitative histomorphometry, whose objective is to achieve the concrete spatial description of the entire tumor morphology and invasive elements (nuclei orientation, texture, architectural shape) based on standard images obtained by H&E coloration (hematoxylin and eosin) [42].

Frequently, pathologists are interested in identifying a nuclei subset from a particular anatomical region (e.g., tumor nuclei in *lamina propria* in T1 bladder cancer [43]; the ratio between Ki67-positive tumor nuclei and total tumor nuclei in neuroendocrine tumors of the breast or pancreas [44,45]; and centroblasts; presence in the follicles in follicular lymphoma, etc.). There is therefore a growing interest in developing algorithms that can identify with great precision a subset of cells in a particular anatomical region [46,47], with many methods being proposed in this regard in recent years.

Xu et al., in 2011, proposed an algorithm based on GAC models (Geodesic Active Contours) [48], which uses mean shift clustering and normalized cuts strategies to identify general histological structures; the algorithm begins from a color sample, usually small—a few pixels on the object of interest—to identify all the objects on the image that match the original color sample, generating an initial outline for the GAC mode. Then, it uses an edge-detection function on the GAC model based on a local structure tensor-based color gradient, obtained by calculating the local min/max variations contributed from each color channel. The method has been successfully used in the segmentation of glandular regions in prostate biopsies.

Subsequently, deep-learning algorithms were proposed to generate a probabilistic map of regions with and without nuclei from the investigated image, created on the basis of previous knowledge. Song and collaborators, in 2015, used a multiscale convolutional network to generate this map [49]. Xing and collaborators, in 2016, proposed a region increasing based on the topological analysis of the probabilistic map, in order to segment the individual nuclei [50,51]. Unfortunately, such methods require learning algorithm re-training in order to process new images and are not reproducible on images coming from organs other than those on which the initial training was performed. A possible solution is to train the algorithms for nuclei segmentation on images from different organs.

Kumar and collaborators [40] developed a database of 30 WSI images of tissue samples from multiple organs, taken from 18 hospitals and included in The Cancer Genome Atlas, using different slide preparation protocols; over 21,000 nuclei were manually annotated and used to train the deep-learning algorithm; the nuclei segmentation was formulated as a problem of identifying three classes, with the nuclei edges being the third class in generating the probability map. Mahmood and colleagues [52,53,54] adapted a generative model to achieve nuclei segmentation on images from four different organs and generated synthetic images that were combined with real ones to train a convolutional neural network for nuclear segmentation.

Zarella et al., 2017, proposed a fast algorithm for nuclei identification and segmentation based on the analysis of color, shape (surface, proportions, circularity) and grouping, to demarcate the individual nuclei and to segregate those overlapped. It was adapted specially to process high-resolution histological images prepared through H&E staining [16]. Li et al., 2018, developed a multistage deep-learning algorithm for mitosis detection in breast biopsies, prepared through H&E staining [55].

Cell counts: The manual interpretation of histological images involves extremely laborious tasks, such as cell counting. These quantitative assessments are not exhaustive, as they consider only particular regions from biopsies (so-called “hotspots”—points of interest) and specific anatomical regions [56]. Computer-aided diagnosis offers increased efficiency, precise quantification, and advanced functions for histopathological image analysis and interpretation, decreasing pathologist workload as well as observation inter-variability and intra-variability. Most current research in the analysis of digitized histological specimens automated refers to deep-learning [57].

The deep-learning techniques combined with WSI mimic human visual acuity and perform the exhaustive analysis of the entire tissue specimen; due to the high volume of processed information and the algorithms complexity, however, research tends to focus on narrower objectives that require the analysis of smaller images areas, such as mitosis detection, anatomical region identification or tumor process identification [1,58,59].

Many valuable results were reported; a few examples are:

- Detection of ductal carcinoma in situ on H&E-stained WSI biopsies [60].
- Detection of tumor regions in neuroendocrine pancreatic tumors [43].
- Development of a new adaptive sampling method for WSI based on the Monte-Carlo technique [61].
- Developing a new method for region-of-interest selection in breast cancer that minimizes the data transfer [28].
- The TissueMark™ platform (Philips Pathology, The Philips Centre, Guildford, Surrey, UK) is used for the automatic annotation of tumor outlines and the evaluation of tumor cell percentage—this provides better results than the manual evaluation [62].

Tissue identification and classification: It is performed for diagnosis purposes, since there are pathologies (e.g., cancer) that entail alterations in tissue architecture and nuclear morphology. Therefore, there has been a need to develop algorithms for automatic tissue classification and disease grading, which can be applied as diagnostic tests to assess the disease’s aggressiveness degree. This can be used to predict the evolution and customize treatment, through precision medicine [42]. Specific features are tracked and quantified, then classified into two categories:

a. Handcrafted characteristics: They are described by measurable attributes of the image, whose interpretation is known. They are of two types: general attributes, which can be evaluated regardless of the tissue’s nature and the investigated pathology (e.g., the nuclei shape and size, the tissues texture and architecture), and domain-dependent attributes, which are specific to a particular organ or pathology. The investigation techniques include wavelet processing, which is used to characterize textures, and graph-based approaches (e.g., Voronoi and Delaunay tessellations, minimum spanning trees, cell cluster graphs), for spatial characterization.

b. Unsupervised features: These refer to less intuitive image attributes that have been identified as significant through the automatic processing of certain training image libraries using machine learning. They are useful for the automatic modeling and differentiation of pathological processes, but they are more difficult to interpret and justify for the pathologist. Parent–child-type relations are assigned between the morphological elements of interest, on the basis of which automatic classifiers using machine learning techniques are created—their usage consists of quick querying and manipulation through embedded commands or scripts [63].

Cell/morphological profiling: This is a relatively new field based on image analysis, which aims to quantify phenotypic differences between different cell populations and to identify chemical and genetic changes in biological systems [64]. Cell populations are described by hundreds of morphological features, which are measured comparatively in the control sample and the active sample and treated with various chemical and biological perturbagens in order to identify similarities, matches or anti-correlations between different treatment conditions. Thus, it becomes possible to identify disease-specific phenotypes, gene and allele functions, and the targets or mechanisms of action of drugs.

The workflow in cellular profiling consists of eight stages:

(1) Image analysis: specific measurements (features) are extracted from digital images to describe each cell’s status in terms of shape, color, texture, microenvironment, and context; as many features as possible are recorded in order to maximize the chances of detecting changes under the action of external factors;
(2) Image quality control: this is carried out with statistical methods in order to eliminate the images or areas affected by artifacts: blurring, saturated pixels, atypical cells/outliers;
(3) Preprocessing of extracted features: elimination of absent values, correction of plateau and batch effects, normalization;
(4) Size reduction: irrelevant features are eliminated, and the similar ones are joined in order to reduce redundancies, through statistical methods: calculation of correlations in compliance with the principle of “minimum redundancy–maximum relevance”, linear transformations, PCA (principal components analysis), factorial analysis and discrimination analysis;
(5) Aggregation of individual data through vector representations at the population level, which summarize its typical features. Statistical methods are also used—simple aggregation by calculating the average, median or KS profile, or sub-population identification by clustering and classification;
(6) Similarities between profiles measured based on distances and the concentration effect quantification—such an effect occurs in the case of chemical perturbagens, tested at different concentrations;
(7) Sample quality evaluation;
(8) Downstream analysis, to interpret and validate the patterns identified in the morphological profiles, by hierarchical classification, visualization (data projections—PCA, Isomap, tSNE t-distributed stochastic neighbor embedding) and data/methods sharing in the scientific community. Examples of software developed for cell profiling are: CellProfiler and EBImage (open source), Columbus and MetaXpress (commercial solutions), Cytominer (package of function in R for morphological profiling) or Python and MatLab (for processing with specific algorithms).

Investigations to discover new clinico-pathological correlations: Over time, many important discoveries in medicine have been made by anatomo-pathologists through the microscopic investigation of specimens; for example, *H. pylori* was discovered by investigating the gastric mucosa in patients with gastritis, as well as tumor staging, with major implications for designing treatment plans and making accurate prognoses about the patient’s evolution. Currently, due to advances in medical imaging generally and in digital pathology particularly (WSI techniques), a large amount of imagistic data is available to researchers, but they are practically impossible to investigate manually through visual inspection. The only solution is their automated processing through machine learning, in order to detect new clinical–pathological correlations.

A particular case is represented by genomic technologies, applied at the DNA level, transcriptomics, and proteomics, which facilitate the in-depth analysis of malignancy degree and the identification of new subclassifications, allowing us to forecast more precisely the disease’s evolution and the response to treatment.

It has been established that the changes at the molecular level in genetic expression are correlated with structural and vascular phenotype changes, identified through imagistic investigations; that is, we are dealing with a fusion between radiology and pathological anatomy, materialized through the spatial alignment of radiological in vivo images with histological ex vivo images, in order to map the area of pathology extension between the two types of investigation and to identify radiological markers correlated with the histo-morphometric tissular changes. This allows us to better characterize the disease (radiogenomic studies) and possibly to highlight new protein–genomic associations and create improved molecular classifications. The difficulty of this approach lies in the precision required to align the two types of investigations and to establish the spatial correspondence between them; a possible solution is the 3D reconstruction of histological specimens based on 2D digital images, which will correlate with the 3D reconstructions of similar radiological images [42].

Computerized processing combined with image registration is a relatively new approach that allows:

- The study of different biomarker behavior inside the same cell [65,66];
- A better understanding of the tumor microenvironment—the types of cells that border each other, their heterogeneity and number, evaluated through statistical methods. This allows us to identify the tissue composition and the phenotypic signature (in colorectal cancer) [67];
- The evaluation of evolution forecast based on histopathological images combined with genomic biomarkers (in glioma, pulmonary adenocarcinoma, or liver cancer) [68,69,70];
- The evaluation of evolution forecast and sensitivity to chemotherapy treatment in breast cancer through the combined study of genetic expression, copy number alteration and histopathological images (FusionGP) [71];
- The anticipation of somatic mutations;
- The discovery of new genetic combinations responsible for certain pathologies (e.g., autoimmune thyroiditis) [72], etc.

Another example is the localization and quantification of immune cell infiltrates. The T cell amount and location are the basis of the Immunoscore™ technique (Laboratory of Integrative Cancer Immunology INSERM, Paris, France) in colorectal adenocarcinoma: the patients with a low density of CD3+ and CD8+ T cells in the tumor center and invasive borders have an increased risk of disease relapse [73], so this is a useful marker in deciding the treatment plan.

Proteomics data provide molecular features with diagnostic value, which accurately describe certain biological processes in cancer, and recent studies have shown a clear correlation between them and the interpretation of histopathological images. The analysis of proteomic data was performed using a random forest classifier to discriminate between the control group and cancer cases, and the imagistic data were processed with a deep-learning algorithm based on convolutional neural networks [74].

Biomarker Evaluation for Diagnostic Purposes: Currently, pathological evaluation by traditional methods is not enough to sustain large-scale tissular biomarker studies based on a high-precision, reproducible and objective analysis, correlated also with the clinical aspects [75,76]. Digital pathology provides new diagnostic facilities and research opportunities based on the development of analysis techniques specific to large images—“big data” [77], which “incorporate” fundamental prognostic data into their content [78].

Accurate quantitative models of the disease are produced (on mathematical bases), that allow us to predict the aggressiveness degree correlated with the patient’s diagnosis [42]. Built-in cell segmentation algorithms and WSI techniques allow us to measure the cell morphology and the biomarker expression and can assist in cell-type classification according to certain features, providing a comprehensive phenotypic description of each cell within the tissue sample. A quantitative cellular map of the entire tissue specimen is obtained, which can be further selected, interrogated, or filtered, allowing us to discover morphological subtleties that are not immediately visible in the case of classical evaluation. All these results can be obtained regularly in only a few minutes, without it being necessary to use specialized hardware devices. Many results in this direction, obtained through mathematical or statistical approaches, are reported in the scientific literature; some of the most recent examples are:

Prognosis of clinical-pathological subtypes in breast cancer by Fisher discrimination analysis of ER, PR, HER-2, and Ki-67 expression and radiomic features extracted from DW (diffusion-weighted) images obtained through MRI (radiomics is the process of converting digital medical images into sets of multi-dimensional data, and is involved in diagnosis, cancer detection, disease grading and prognosis/response to treatment evaluation) [79];

Evaluation of hormone receptor response in breast cancer through an ER and PR expression investigation and automated quantification; a reactivity score is assigned based on WSI image staining and surface analysis using the Allred Scoring method. The reported correlation coefficients between the automated evaluation and the one carried out by human experts are 0.881—overall, and 0.922 for the ER sections and 0.840 for the PR sections, respectively, when taken separately [80];

Evaluation of the treatment response and the survival forecast in breast cancer by calculating the RCB index (Residual Cancer Burden) using a DCNN system (Deep Convolutional Neuronal Network). The RCB index is calculated using six parameters: the primary tumor bed area (length and width), the overall cancer cellularity, the percentage of in situ cancer, the number of positive lymph nodes, and the diameter of the largest metastasis. The reported correlation coefficient between the automated evaluation and the one carried out by human experts is 0.82, while the correlation between the evaluations of two human experts is 0.89 [81].

Among the parameters used to calculate the RCB index, the most important is the cancer cellularity estimation, defined as the proportion of cancer within the residual tumor bed; its manual calculation is laborious, requires experience and is affected by a high degree of inter-assessor variability. Pei et al. proposed in 2019 an automatic procedure to calculate this index, based on deep feature representation, tree boosting, and support vector machine (SVM), avoiding nuclei segmentation and classification. The correlation coefficient reported by the authors between their method and the evaluation made by the human expert was 0.94 [20].

Evaluation of dystrophin production as surrogate marker for treatment efficacy in Duchenne muscular dystrophy, based on the staining intensity and the percentage of biomarker-positive muscle fibers in tissue cryosections, using the MuscleMap algorithm (Flagship Biosciences, Westminster, Colorado) [31].

Improvement of practices in immuno-oncology and immuno-profiling: The detailed investigation of the tumor microenvironment, identification and quantification of the immune cell population, as well as their spatial location and the expression of immunological markers, are compulsory when characterizing a patient’s clinical evolution and choosing the optimal, individualized treatment scheme. In this regard, digital pathology provides valuable precision tools: automatic evaluation (digital scoring) of the immune control points inhibitors expression (e.g., PD-L1), spatial analysis of tumor immune cell infiltration, and the multi-layered analysis of biomarkers in interdependence. This is possible through image registration and virtual multi-staining or the integration of digital information about ICI (Immune Cell Infiltration) with molecular data, for a better configuration of the cancer immunogram and individualized prognosis regarding the response to immunotherapy [82].

Reports standardization and new features addition: These are necessary to eliminate the inherent degree of subjectivity that characterizes the assessment carried out by the pathologist. A concrete example is the interpretation of ER (Oestrogen Receptor), PR (Progesterone Receptor) and HER-2 (Human Epidermal Growth Factor Receptor 2) staining standardization, in breast cancer [83,84]. Currently, the FDA (USA—Food and Drug Administration) and CE IVD (Europe—In Vitro Diagnostics) approve a relatively small number of applications for the automatic evaluation of pathological features, such as the expressions of ER, PR, HER-2 and Ki-67 in breast cancer, and they have similar results to those obtained by a human evaluator [63,84].

Integration of histopathological analyses with other clinical data: For a precision diagnosis, the analysis of histopathological images should be correlated with the patient’s other clinical data (demographic data, medical history, laboratory test results, medical reports, etc.). These data are often available as unstructured and non-standard reports, from which the relevant information must be extracted through natural language processing techniques. Such techniques can also be implemented using deep-learning algorithms, which allow for filtering information from disparate sources and highlighting the subtle connections between them. This is helpful for the pathologist to take the best clinical decision.

Among the main producers of digital pathology solutions, we can mention Siemens (Syngo Carbon and Concentriq Dx^3^ software—an integrated system for imaging data management), Zeiss (In Vivo Pathology Suite Convivo—a solution for digital image consultation with real-time feedback on tissue microstructure), Roche Diagnostics (uPath Enterprise software, with dedicated applications to identify Ki-67-positive stained tumor cell nuclei, PD-L1-positive and -negative tumor cells, HER2 gene status, ER- and PR-positive and -negative tumor cells and others), Philips (IntelliSite Pathology Solution—integrated platform for assistance of all stages in the pathology process), Sectra (Sectra Digital Pathology Solution—a system for primary diagnostics in pathology), Proscia (Concentriq Digital Pathology Platform—a flexible architecture which also integrates AI applications, like Automated QC—for performing quality control on images of H&E stained slides and DermAI—for classifying of dermatopathology slides), 3DHISTECH (solutions for diagnostics, research and education: Pannoramic Pathology Diagnostic System, PathoNet, ESchool), and Bosch (AI-powered Digital Pathology Solution). The list is open and constantly updated.

### 4.2. Education and Training

AI applications combined with WSI are useful in training programs because they provide on request quality standardized digital images available for sharing that are easy to handle (crop, pan, zoom) and to annotate in real time [4,6]. There are also training applications that provide automatic assistance and direct visual feedback on the carried-out evaluations, contributing therefore to workflow standardization in evaluating the usual and atypical features of anatomic-pathological images, and to the concordance level increasing between investigators.

The WSI technique is often used in virtual conferences, seminars and workshops, presentations, discussion groups and case studies [6]. Synthetic images, digitally generated, are also used for dynamic training in real time; the pathologists can efficiently improve their skills and have the opportunity to better understand the difficulties they may face [1].

Nowadays, different categories of online resources are available for public access, assisting the pathologist in his activity, although the demand for such resources is much higher and varied than the supply:

- Libraries for digital image management, e.g., OpenSlide [85], Bio-Formats [86], NEPTUNE, CureGN [87]. The DPR (Digital Pathology Repository) concept is being used more and more frequently—this is a new and inexpensive way to organize resources, for the long-term storage of high-resolution WSI histological images, in Web-hosted imaging libraries, available online, without geographical boundaries and with significant time savings [87];
- Software to crop WSI images, to extract patches and to analyze them, e.g., SlideToolKit [88], ImmunoRatio [89], Visiopharm [63];
- Open-source image analysis tools: ImageJ Image Process and Analysis in Java [90,91], NIH Image [74], Fiji [92], Icy [93], CellProfiler [94], QuPath Open-Source Software for Quantitative Pathology [10];
- Web platforms for data management and collaborative analysis, e.g., Cytomine [95].

Each of these tools provides a valuable contribution, but pathologists still need specific tools to assist their work, e.g., forecast modules allowing them to develop new digital pathology algorithms [88]. Thus, researchers without access to expensive solutions continue to carry out the laborious manual evaluation of digitized images, with limited reproducibility [96], or use automated tools with restricted functions (for image sampling, cropping and elements of interest extracting or specific quantitative analysis).

Some of the existing software provide pre-established, easy-to-use algorithms for routine analysis in pathology, but they also offer the possibility to implement “building blocks” in order to create new, interconnected and customized modules [10,97]. It is thus possible for the researcher teams to create and to add their own image processing algorithms that, combined in an efficient manner with the existing tools—albeit limited (MATLAB, etc.)—can facilitate the development of new automated classification and/or forecast models [10].

### 4.3. Quality Assurance

Digital images stored in laboratory information systems or in Intranet local networks facilitate quality assurance and monitoring, diagnostic error prevention and risk reduction for patients, through teleconsultation, the surveillance of inter- and intra-observer variance, proficiency tests and more effective methods for image archiving [1].

Histopathology is a medical specialization incorporating activities that cannot be carried out remotely by their nature, e.g., surgical and cytological specimen handling and preparing. Instead, tele-pathology applications combined with the WSI technique provide the optimal framework for remote investigation and diagnosis, without requiring the physical transport of slides and without geographical restrictions. In addition, collaboration between specialists, access to a “second opinion” and interdisciplinary studies are facilitated, with direct effects on the quality of the medical act.

A study carried out during the COVID-19 pandemic demonstrated the usefulness and addressability of tele-pathology applications, which proved to be a viable solution under the urge to keep physical distance; about 75% of the interviewed specialists claimed that they used tele-pathology platforms in daily work, for quick diagnosis and cases sorting, and 66.7% claimed to use them to request a second opinion. The study also revealed a 25% increase in the use of digital tools for diagnosis and documentation, allowing time savings [98]. Consequently, the FDA has approved the use of tele-pathology systems for screening, primary diagnosis, second opinion and training; such systems can be implemented statically (the images are recorded, stored and then transmitted to be viewed and analyzed remotely), but also dynamically (live video images are transmitted and viewed remotely in real time) and even robotized dynamically (the investigator remotely controls the microscope to retrieve images)—such practices are used in cytology and surgical pathology [87].

Anatomo-pathologists can easily improve their competence level by being permanently informed about the new results in the field and the most advanced diagnostic tools.

Artificial intelligence tools can be implemented as auxiliary tools in the Computer-Aided/Computer-Augmented Diagnosis workflow, as prospective or retrospective control tools for diagnostic accuracy and to prevent the human errors. They are being used more and more frequently together with the classical slides, and the dynamics of achievements will imprint a paradigm shift in both microscopic and clinical morphological research [99,100].

## 5. Advantages Brought by Digital Pathology

Some of the main benefits of integrating digital pathology into the current medical practice are the following [2]:

### 5.1. Reducing the Risks for Patients

The use of digital pathology-integrated systems based on the electronic transmission of images to the pathologist reduces the risk of wrong labeling or mixing of specimens by a percentage between 13 and 99%. The estimated incidence of such errors in classical pathology laboratories is about 1%. Additionally, the risk of losing tissue samples or slides, which do not degrade or deteriorate over time, is also significantly reduced.

### 5.2. Workflow Optimization

Digital systems provide flexibility in allocating cases to physicians, according to the overall workload in the laboratory. In addition, the physicians will have the freedom to choose the cases they are going to take over for analysis and to request colleagues’ collaboration when a second opinion is needed. Clinical cases can be easily traced throughout the laboratory; they can be retrieved immediately if necessary and archived efficiently, and the efficiency in diagnosis can thus be improved by up to 13%. It also becomes easier and faster to perform measurements and annotations on images, and the time required for transferring slides from the ward where they are prepared to the analyst and back is eliminated. The urgent cases, which require priority in the diagnostic process, can be quickly identified and labelled as such. Information systems also facilitate inter-departmental collaboration between histopathology and nuclear medicine services when it is necessary to transfer images for mixed analysis.

### 5.3. Improving the Quality of the Working Environment

Digital systems allow us to implement a more flexible work schedule, eliminating the need for the pathologist to physically go to the laboratory where the slides are located, thus giving him more freedom in organizing his time. Training programs become easier to attend and more valuable in content, since they facilitate the access to rare, unusual, or instructive clinical cases; the teacher can also communicate faster, with several students simultaneously, and receive instant feedback from them. The use of advanced IT tools makes the field more attractive for young people, with histopathology being perceived as a modern, innovative, and dynamic specialization.

### 5.4. Improving the Quality of Services

This is the direct consequence of simplifying information transfer and review, especially when a second opinion is requested. Access to the images archive is substantially simplified, as is the simultaneous analysis of several images for their synchronized evaluation, the accurate investigation of features with diagnostic value, and the regions of interest annotation, which can be used later for audit. The implementation of quality and extensive imaging databases stimulates classical medical research and the development of new algorithms for fast diagnosis or realistic forecasts.

There are studies that have proposed the organization of digital pathology services as remote services [101] and have investigated the effectiveness of such an approach, reporting notable results: an average digital image transfer speed of 20 Mbps; an amount of low-quality images for which rescanning was necessary of 1%; a 99% concordance between the diagnosis established by classical and digital microscopy; a decreased time required by the digital image evaluation, correlated with a higher percentage of cases for which the diagnosis was transmitted in less than 3 days; a total concordance between the physicians’ opinions when the images were reviewed by a second specialist; and a high level of satisfaction for all the categories of professionals involved in the project.

It is also recommended to integrate digital pathology services into the laboratory information systems already implemented in the pathological anatomy department [102], in order to optimally streamline the information circuit and to facilitate the storage in common, centralized databases.

## 6. Limits in Digital Pathology

Many techniques of automated processing, and particularly the deep-learning-based systems, are disputed because they do not explain how they reach the final results. This must be clearly argued from a clinical and forensic point of view. Therefore, the current research is oriented towards providing a degree of transparency to automated processing algorithms, in order to make it easier to interpret and argue their conclusions. Some pathologists are cautious in adopting the new technologies, considering that such techniques limit their control over their own work, which becomes public and is thus more exposed to deterioration, loss, and anonymous criticism [87]. There are cases when the whole WSI technique is regarded as disruptive. Staining standardization procedures are not yet extensively used in practice as there still are important differences between laboratories for most immunohistochemical staining procedures, especially for those where the intensity is essential in evaluation (e.g., Ki-67); this makes collaborative projects difficult. The specimens’ full processing and the extraction of all available data require significantly increased computing power and storage space, also increasing the risk of erroneous information due to possible artifacts. Additionally, statistical processing errors (multiple testing or subjective interpretations) must also be avoided, so the automated analysis of digital images must only be carried out rigorously by specialists competent in the field with complete and adequate scientific documentation, while regarding all quality standards [82].

Legally, the new GDPR regulation adopted in the EU stipulates that “the data subject shall have the right not to be subject to a decision based solely on automated processing”, with significant medical implications added to the financial and economic implications of the automated processing instruments using for anatomopathological diagnosis.

The best solution is combining the specialist physician’s expertise with the automatic processing tools in order to transform the traditional qualitative assessments into more accurate, consistent and useful quantitative analyses, whether semi-automated (with the partial intervention of the human operator), or automated [81,103]. This is because traditional methods are affected by some degree of imprecision, e.g., the concordance coefficients between the results offered by different pathologists on the same tissue specimen may vary from 0.86 to 0.95 [21]. Automated analysis and predictive modeling algorithms allow us to identify and characterize tissular regions of interest, individual cells, or structures and to classify them based on relevant features (biomarkers levels of expression, morphometric parameters, localization) [63,104,105]. Meanwhile, the physician retains the task of summarizing and correlating the obtained results, in order to adopt the best decision for the patient.

This interdisciplinary specialization, which combines histopathology with computer tools, is currently in full development. Computer-aided analysis, detection and diagnostic tools for high-resolution digital histological image processing are still in the process of implementation and optimization [48,106].

## 7. The Future in Digital Pathology

In our opinion, the future in digital pathology belongs to AI tools. The scientific literature offers more and more articles in this direction of research, revealing the fact that AI technology represents a recommended solution in this particular field for multiple reasons. The most significant one is that high-quality tissue slides contain a large amount of information (up to 10 gigapixels), which is far too much to be fully processed by human specialists but is suitable for automated tools [107,108]. Such tools are not intended to replace the human experts, but only to assist them in their work and to improve their skills and expertise. In this regard, very interesting results are presented by Berbis et al. [109], who conducted a forecasting study about the role of AI in pathology within the next decade. They interviewed 39 experts in the field from the whole world (USA, Europe, Canada, Japan and New Zealand); all experts agreed that AI will improve diagnostic accuracy and standardization, particularly the detection of rare events (such as tumor metastases) and tumor grading, making histopathologic analyses more quantitative. Furthermore, they predicted that specific AI applications will be routinely used by 2030, while the tasks routinely performed by pathology technicians will be significantly modified, with the basic tasks being performed automatically. Regardless, advanced analysis and forecasting will still be performed by human experts. There are still significant issues to be solved (technical, regulatory, and financial) before reaching the full potential of AI for the pathology field. An emerging approach, which seems to have a large potential, is represented by Interactive Machine Learning; this approach eliminates the concept of the “black box”, is specific for most AI algorithms and empowers human experts. The pathologist interacts with the machine during the diagnosis process and is able to control and guide it in feature extraction [110]. There are no doubts that we are facing a very vivid research field, and the future will offer spectacular surprises.

## 8. Conclusions

In digital pathology, computer-aided image analysis provides superior accuracy, reproducibility, and standardization in research based on microscopic morphology, compared to classical histological examination, since the amount of information contained in the cell exceeds what can be observed by the human specialist alone. Computer-aided image analysis has a superior potential to identify, extract, and quantify features in more detail compared to the human pathologist’s evaluating possibilities; it performs tasks that exceed its manual capacity, and can produce new diagnostic algorithms and prediction models applicable in translational research. Thus, it is able to identify new characteristics of diseases based on changes at the cellular and molecular level. Despite the reluctance of regulatory agencies so far to approve the using of scanned images for primary diagnosis, computer-assisted analysis and digital pathology will become basic tools in making clinical decisions in the near future.

## Figures and Tables

**Table 1:** Synthesis of search results by different keywords.

Subject (Keywords)	2017	2018	2019	2020	2021	2022	Total
general topics/techniques							
computer-assisted diagnosis/ computer-assisted interpretation		2	3	3	2		10
image analysis software/ digital image analysis/ computerized image analysis	8	15	16	21	30	9	99
Whole-Slide Imaging/WSI	43	57	82	96	129	22	429
applications in clinical diagnosis							
color variation/ color normalization/ stain normalization/ spectral normalization	3	1	6	9	11	6	36
segmentation	16	9	38	39	67	12	181
counting	4	5	7	8	12	2	38
region of interest	2	3	1	2	6	1	15
profiling	2	5	5	12	14	3	41
feature extraction	1	5	1	7	11	1	26
genomic/proteomic/phenotype	5	8	9	7	14	5	48
biomarker	15	12	5	29	47	7	115
standardization	5	2	7	7	8	2	31
quantitative image analysis	1	2	4	4	4		15
morphometry	2	1	2	3	1		9
other applications							
information system	2	3	3	3	5	2	18
education	9	11	13	21	29	3	86
training	21	19	49	62	99	22	272
quality control	2	5	5	4	8	3	27
IT methods—machine learning	32	66	151	265	439	110	1063
artificial intelligence	1	13	35	72	132	34	287
machine learning	12	18	40	75	106	27	278
deep learning	14	28	59	98	165	43	407
convolutional neural network	5	7	17	20	36	6	91
IT methods—statistical approaches	10	14	13	23	25	7	92
component analysis		1		1	1		3
clustering	1	4	5	8	6	2	26
mixed model	1						1
similarity measures	8	9	8	14	18	5	62

## Data Availability

Not applicable.
